# Evaluation of the Impact of Letrozole in Combination with the GnRH Antagonist Ovarian Stimulation Protocol in Patients Expected to Have a Poor Ovarian Response (POSEIDON Groups 3 and 4)

**DOI:** 10.3390/medicina60030407

**Published:** 2024-02-28

**Authors:** Bulut Varlı, Yavuz Emre Şükür, Eda Üreyen Özdemir, Batuhan Özmen, Murat Sönmezer, Bülent Berker, Cem Atabekoğlu, Ruşen Aytaç

**Affiliations:** 1 Department of Obstetrics and Gynecology, School of Medicine, Ankara University, 06620 Ankara, Turkey; yesukur@ankara.edu.tr (Y.E.Ş.); batuhanozmen@ankara.edu.tr (B.Ö.); msonmezer@ankara.edu.tr (M.S.); berker@medicine.ankara.edu.tr (B.B.); cematabekoglu@ankara.edu.tr (C.A.); raytac@ankara.edu.tr (R.A.); 2 Department of Obstetrics and Gynecology, Ministry of Health Ankara City Hospital, 06800 Ankara, Turkey; eda.ureyen@gmail.com

**Keywords:** letrozole, GnRH antagonist, live birth rate, poor responder, POSEIDON

## Abstract

*Background and Objectives*: The objective of this study was to evaluate the impact of adjuvant letrozole administration during ovarian stimulation using the gonadotropin-releasing hormone (GnRH) antagonist protocol on treatment outcomes in women categorized into POSEIDON groups 3 and 4. *Materials and Methods*: This retrospective cohort study analyzed data from patients classified into POSEIDON groups 3 and 4 who underwent fresh embryo transfer subsequent to intracytoplasmic sperm injection following a GnRH antagonist stimulation protocol between January 2017 and December 2021. Patients were divided into two groups: the GnRH-LZ group, who received letrozole at a dosage of 5 mg/day for five consecutive days, and the GnRH-ant group, who did not receive adjuvant letrozole. The primary outcome measure of the study was a comparative analysis of live birth rates between the two groups. *Results*: A total of 449 patients were deemed suitable for final analysis and were allocated into two groups: 281 patients in the GnRH-ant group and 168 patients in the GnRH-LZ group. Live birth rates were found to be comparable in both groups (11% vs. 9%, *p* = 0.497). Letrozole administration significantly reduced the total amount of gonadotropins required (2606.2 ± 1284.5 vs. 3097.8 ± 1073.3, *p* < 0.001), the duration of ovarian stimulation (11.2 ± 3.9 vs. 10.2 ± 3, *p* = 0.005), and the serum peak estradiol concentration (901.4 ± 599.6 vs. 463.8 ± 312.3, *p* < 0.001). *Conclusions*: Adjuvant letrozole administration did not demonstrate a significant impact on live birth rates among women categorized into POSEIDON groups 3 and 4. However, this approach may offer potential cost reductions by diminishing the necessity for exogenous gonadotropins and shortening the duration of ovarian stimulation.

## 1. Introduction

A poor ovarian response (POR) to controlled ovarian stimulation (COS) represents a critical factor influencing the success of assisted reproductive technology (ART) treatments. Despite the well-established impact of POR on treatment outcomes, its definition has long been a subject of controversy. A meta-analysis encompassing 47 studies involving patients with POR revealed a staggering array of 41 different definitions, each employing distinct criteria and parameter values [1]. In 2011, the European Society of Human Reproduction and Embryology (ESHRE) introduced the Bologna criteria (BC) to mitigate diagnostic inconsistencies [2]. According to the BC, POR is characterized by the presence of at least two of the following criteria: (i) a prior insufficient response, defined as the retrieval of ≤3 oocytes during standard COS; (ii) abnormal ovarian reserve, indicated by an antral follicle count (AFC) of <5–7 follicles and/or anti-Müllerian hormone (AMH) levels of <1.1 ng/mL; or (iii) maternal age exceeding 40 years. Despite the establishment of the BC, only 53% of studies involving POR patients utilized these criteria for defining POR in 2017, a lower than anticipated adoption rate in its fifth year [3]. The BC has drawn criticism for several reasons, primarily for grouping patients with differing prognosis under a single definition. Additionally, the use of a 40-year age cutoff to assess the impact of female age on oocyte quality has been deemed insufficiently evidence-based [4]. In 2016, the Patient-Oriented Strategies Encompassing IndividualizeD Oocyte Number (POSEIDON) group, comprising reproductive health professionals, introduced a novel and comprehensive classification system. The primary objective was to alleviate the heterogeneity observed in the BC-defined population and devise personalized treatment protocols for these individuals. The POSEIDON classification stratifies patients into four subgroups based on age, ovarian reserve markers, and prior ovarian response (oocyte yield) during COS, if available. In practice, the POSEIDON classification delineates patients with POR into two categories: ‘unexpected POR’ (groups 1 and 2) and ‘expected POR’ (groups 3 and 4) [5]. Upon comparing these groups in terms of cumulative live birth rates, the highest rate of 22.6% was recorded in group 1, with this rate progressively declining to 4.8% in group 4 [6]. Given the notably low live birth rates (LBRs) in POSEIDON groups 3 and 4, numerous treatment modalities are recommended for these patients [4], albeit some lacking approval from the US Food and Drug Administration (FDA) for use with POR patients.

Letrozole exemplifies the off-label use of an aromatase inhibitor (AI) in infertility treatment [7]. Aromatase inhibitors function by reducing circulating estrogen levels, primarily by inhibiting the aromatization of androgens to estrogens, mainly in the granulosa cells of the ovary. This inhibition leads to a decrease in estrogen levels, which can affect the pituitary–gonadal axis and the uterus in various ways. Pituitary gonadotropin secretion increases due to the loss of the central negative feedback effect of estrogen on the pituitary gland. At the same time, intraovarian androgen concentration increases due to inhibition of aromatization and this results in a potentiation of the follicular response to follicle-stimulating hormone (FSH) [8]. Additionally, letrozole may impact endometrial receptivity by suppressing supraphysiologic estrogen levels during COS, thereby reducing embryo–endometrial asynchrony and, consequently, influencing implantation and miscarriage rates [9]. Moreover, a decrease in estrogen levels reduces the suppression of pituitary luteinizing hormone secretion, leading to an increase in progesterone secretion during the luteal phase [9]. These effects of letrozole may contribute to improved assisted reproductive technology (ART) outcomes.

Letrozole was initially employed by Mitwally and Casper for poorly responding patients [10]. Subsequently, a randomized controlled trial (RCT) was published in 2004 [11] and following investigations by other researchers have examined the effect of letrozole [12,13,14,15,16,17,18,19,20]. In 2017, a Cochrane meta-analysis was conducted on a topic with a substantial volume of literature. The analysis indicated that letrozole reduced the total amount of gonadotropins used during COS. However, no improvement in clinical pregnancy rate (CPR) or live birth rate (LBR) was observed [21]. On the other hand, a recent meta-analysis by Qin, conducted three years after the publication of the Cochrane review, indicated that the addition of letrozole to the gonadotropin-releasing hormone (GnRH) antagonist treatment protocol resulted in a decrease in the total gonadotropin dose needed, as previously noted in the Cochrane review. Additionally, the meta-analysis revealed an increase in CPR, contradicting the findings of the Cochrane review [22]. Importantly, none of the trials included in these two meta-analyses grouped patients according to the POSEIDON classification system.

The aim of this study was to evaluate the efficacy of letrozole in women classified within POSEIDON group 3 and group 4 undergoing ovarian stimulation via a GnRH antagonist protocol.

## 2. Materials and Methods

A retrospective cohort study was conducted at Ankara University, Faculty of Medicine, Department of Obstetrics and Gynecology, Assisted Reproduction Unit, analyzing data from couples seeking treatment between January 2017 and December 2021. The study design received approval from the Institutional Review Board of the Department of Obstetrics and Gynecology on 17 January 2023 (Approval number: 16). Written informed consent is routinely obtained from each couple initiating an assisted reproductive technology (ART) cycle for the anonymous use of their data in scientific studies.

### 2.1. Study Population

Patient charts meeting the criteria for POSEIDON groups 3 or 4 and undergoing a GnRH antagonist treatment regimen with intracytoplasmic sperm injection (ICSI) and subsequent fresh embryo transfer were screened for inclusion. Exclusion criteria comprised the following:

- Female age exceeding 44 years;
- Absence of fresh embryo transfer;
- Presence of structural uterine abnormalities diagnosed via ultrasonography or hysterosalpingography (e.g., dysmorphic uterus, intrauterine adhesions);
- Presence of endocrinological disorders (e.g., diabetes, hyperprolactinemia, thyroid disorders) requiring medication;
- History of recurrent pregnancy loss;
- Use of add-ons other than letrozole during ovarian stimulation;
- Male factor infertility attributed to azoospermia.

Patients diagnosed with expected poor ovarian response (POR) who received adjuvant letrozole during GnRH antagonist cycles were categorized into the GnRH-LZ group, while those not receiving adjuvant letrozole were placed in the GnRH-ant group.

### 2.2. ART Treatment Protocol

Letrozole (Femara, Novartis Pharma AG, Stein, Switzerland) is prescribed at a daily dosage of 5 mg, administered in two separate doses of 2.5 mg tablets, taken 12 h apart. Treatment begins on the second day of the menstrual cycle and is continued for five consecutive days. Simultaneously, ovarian stimulation is initiated with letrozole, using either 225–300 IU/day of recombinant follicle-stimulating hormone (rFSH; GONAL-f; Merck Serono, Bari, Italy) or a combination of 150–225 IU/day of human menopausal gonadotropin (hMG; Menogon, Ferring GmBH, Kiel, Germany; or Menopur, Ferring GmBH, Kiel, Germany) with 150–225 IU/day of rFSH. Throughout ovarian stimulation, the gonadotropin dosage is adjusted based on ovarian response, monitored via transvaginal ultrasound scans and regular measurements of serum estradiol, progesterone, and luteinizing hormone (LH) levels. Upon reaching a leading follicle diameter of 14 mm, cetrorelix (Cetrotide, Merck, Idron, France) is administered at a dose of 0.25 mg as a GnRH antagonist, with treatment continued until the day of final oocyte maturation. The trigger for final oocyte maturation is administered using 250 μg of choriogonadotropin alpha (Ovitrelle, Merck-Serono, Bari, Italy) once at least one or two follicles attain a diameter of ≥17 mm. Transvaginal oocyte pick-up (OPU) is conducted 36 h after the trigger, followed by standard ICSI on metaphase II (MII) oocytes.

Luteal phase support commenced on the day of oocyte retrieval, involving the administration of 300 mg/day of vaginal progesterone (Lutinus 100 mg vaginal tablets; Ferring GmbH, Kiel, Germany). This support was sustained until the 10th week of gestation in women with confirmed clinical pregnancy.

### 2.3. Outcome Measures

The primary objective of the present study was to compare the live birth rates among patients categorized into GnRH-ant and GnRH-LZ groups. Secondary outcome measures encompassed the comparison of various factors including the duration of ovarian stimulation, total amount of gonadotropins used, total number of retrieved oocytes, total number of MII oocytes, number of acquired 2 pronuclear (2PN) embryos, fertilization rate (calculated as the number of 2PN embryos divided by the number of MII oocytes), number of top-quality embryos, implantation rate, clinical pregnancy rate, and miscarriage rate.

The implantation rate for each patient was determined individually using the following formula: the number of gestational sacs divided by the number of transferred embryos, multiplied by 100. Clinical pregnancy was defined as the detection of an intrauterine fetus with cardiac activity during an ultrasound examination at six weeks of gestation. Live birth was defined as the successful delivery of a live infant after at least 24 weeks of gestation.

### 2.4. Statistical Analyses

Statistical analyses were conducted using SPSS Statistics for Windows, version 20.0 (IBM Corp., Armonk, NY, USA). The normality of the data distribution was assessed through histograms and the Shapiro-Wilk test. Parametric tests were chosen accordingly based on the results obtained. Continuous variables were compared using Student’s *t*-test. Pearson’s chi-squared test or Fisher’s exact test was employed to compare categorical variables, as deemed appropriate. A significance level of *p* < 0.05 was considered statistically significant.

## 3. Results

During the specified study period, 449 women from POSEIDON group 3 and group 4 who met the study criteria received treatment. Among them, 281 women (62.6%) underwent treatment using only the GnRH antagonist (GnRH-ant group) protocol, while 168 women (37.4%) were treated with the GnRH antagonist (GnRH-LZ group) protocol combined with letrozole. Letrozole was administered to 31.6% of the 177 women in POSEIDON group 3, whereas 41.2% of the 272 women in POSEIDON group 4 received GnRH-LZ. Notably, in POSEIDON group 4, the proportion of patients treated with letrozole was significantly higher than that in the other group (31.6% vs. 41.2%, *p* = 0.041).

The demographic characteristics comparison between groups is presented in Table 1. Patients in the GnRH-LZ group exhibited significantly higher mean age (36.4 ± 4.9 vs. 34.9 ± 5.1 years, *p* = 0.002), mean duration of infertility (7.5 ± 5.8 vs. 5.9 ± 5.1 years, *p* = 0.004), and mean number of prior IVF/ICSI procedures (1.5 ± 1.8 vs. 1.1 ± 1.4 years, *p* = 0.028). Regarding ovarian reserve markers, women in the GnRH-LZ group demonstrated significantly elevated mean serum FSH levels (13.7 ± 8.4 vs. 11.9 ± 7.5, *p* = 0.022) and a notably lower mean serum antral follicle count (3.4 ± 1.8 vs. 3.9 ± 1.8, *p* = 0.006).

**Figure 1:**
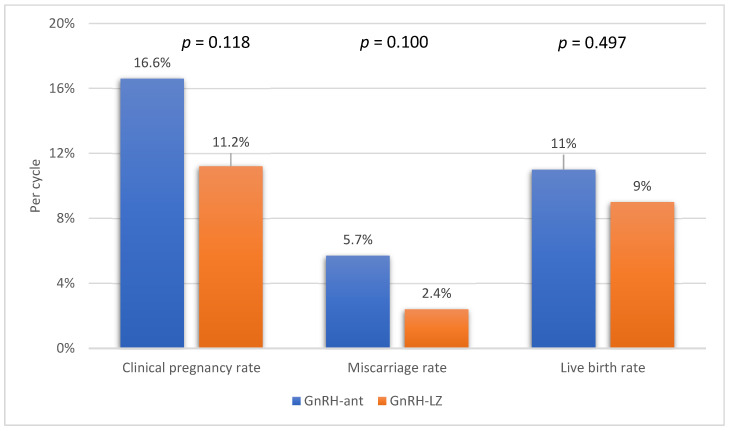
The comparison of clinical pregnancy, miscarriage, and live birth rates between the groups.

Among the entire study population, clinical pregnancy was diagnosed in 66 women (14.6%), with 46 of them (10.2%) achieving a live birth. There were no significant differences observed between the groups in terms of clinical pregnancy, miscarriage, or live birth rates (Figure 1). 

To offer a more comprehensive analysis of the influence of letrozole on treatment outcomes, we conducted separate evaluations of the CPR, miscarriage rate, and LBR among women in POSEIDON groups 3 and 4. However, our analysis did not indicate any advantageous effects of letrozole on reproductive outcomes in either group (Table 2).

## 4. Discussion

Despite one of the initial RCTs investigating the use of letrozole in women diagnosed with POR dating back 20 years, there remains a necessity for studies to assess the impact of letrozole on newly defined populations, given the evolving definition of POR over time [11]. The POSEIDON classification system represents the latest framework for categorizing POR. The objective of the present study was to assess the influence of adjuvant letrozole administration alongside GnRH antagonists on ART treatment outcomes in patients identified as having expected POR based on this recent classification system. The administration of letrozole notably reduced the duration of ovarian stimulation, total gonadotropin usage, and peak serum estradiol levels. However, no significant effects were observed regarding the clinical pregnancy rate or live birth rate.

The existing literature regarding the impact of letrozole administration on assisted reproductive technology (ART) treatment outcomes presents conflicting findings. Ebrahimi et al. [15] and Moini et al. [16] conducted RCTs in close temporal proximity, both involving patients diagnosed with POR according to the BC. Ebrahimi et al. administered letrozole at a dosage of 2.5 mg/day, while Moini et al. utilized a dosage of 5 mg/day. Similar to our study, neither trial identified any statistically significant impact of letrozole administration on the CPR. Furthermore, a Cochrane review conducted in 2017 found no positive effect of letrozole on either CPR or LBR in normoresponders or poor responders [21]. In 2020, a subsequent meta-analysis conducted by Qin [22] included participants from five RCTs and one prospective controlled trial, all exhibiting at least one clinical characteristic of POR, albeit without standardized criteria. The CPR per cycle (RR: 1.57; 95% CI, 1.00–2.44; *p* = 0.05) was higher in the letrozole group compared to the control group, but the CPR per embryo transfer (RR: 1.47; 95% CI, 0.92−2.35; *p* = 0.11) was similar between the letrozole and the control groups. Moreover, the total gonadotropin dosage required decreased significantly (by 529.37 IU; 95% CI, −1207.45 to −111.25; *p* = 0.001) with letrozole administration. However, this meta-analysis did not evaluate the impact of adjuvant letrozole administration on LBR.

Bülow et al. recently conducted the most comprehensive meta-analysis to date on the effects of letrozole co-treatment during ovarian stimulation, encompassing the largest patient cohort [23]. Their analysis revealed that the addition of letrozole to treatment improved the LBR in poor responders by 7% (95% CI, 1% to 13%; *p* = 0.03). However, unlike Qin’s meta-analysis, no significant effect of letrozole was observed on the CPR. In our study, the CPR and LBR in the GnRH-LZ group were comparable to those in the GnRH-ant group. Nonetheless, it is noteworthy that the mean age of patients in the GnRH-LZ group was significantly higher than that in the other group. Advanced female age is recognized as a risk factor for poor reproductive outcomes in ART cycles [24]. Furthermore, it is noteworthy that both the duration of infertility and the number of previous failed ART treatment cycles were notably higher in the letrozole group. Previous studies have demonstrated that these factors can negatively impact fertilization or implantation rates [25,26]. The observation that live birth rates in the GnRH-LZ group were comparable to those in the other group, despite the presence of these aforementioned negative factors, suggests that the positive effects of letrozole treatment, as previously discussed, may have contributed to this outcome. 

Another intriguing finding from our study was the apparent lower implantation rate in the GnRH-LZ group compared to the GnRH-ant group, although this discrepancy did not reach statistical significance. In their study, Kahraman and Tulek [20] noted a beneficial effect of letrozole on the implantation rate among POSEIDON group 3 patients. However, similar to our findings, they also observed a non-significant decline in implantation rate when letrozole was used in POSEIDON group 4 patients. This disparity in implantation rates appears to be attributed to the influence of aging on endometrial receptivity rather than the administration of letrozole [27].

Prior to the publication of two recent meta-analyses by Qin and Bürlow, the American Society of Reproductive Medicine (ASRM) [28] and the European Society of Human Reproduction and Embryology (ESHRE) [29] also provided their guidelines on the administration of letrozole to poor responders. Neither authority recommends routine use of letrozole for patients with POR. The ESHRE guidelines also note that the use of letrozole for ovarian stimulation is off-label and raises safety concerns regarding the potential teratogenicity of letrozole.

The definition of poor POR varied significantly across the studies and meta-analyses mentioned above. In our study, we opted to utilize the POSEIDON classification system for participant selection instead of the Bologna criteria, resulting in a more homogeneous POR population. Kahraman and Tulek [20] also aimed to assess the impact of letrozole administered at a dosage of 5 mg/day for five days with the GnRH antagonist protocol on patients in POSEIDON groups 3 and 4. They observed significantly higher implantation, clinical pregnancy, and live birth delivery rates among POSEIDON group 3 patients who received letrozole, although these effects were not observed in POSEIDON group 4 patients. In our study, separate subgroup analyses were conducted for POSEIDON group 3 and group 4 patients, revealing a trend toward higher live birth rates with letrozole administration in POSEIDON group 3, though the difference was not statistically significant. However, it is important to note that the studies conducted by Kahraman and Tulek included significantly more patients in POSEIDON group 3 than our study, which may have influenced the statistical analysis results. In another study, Lin et al. [30] evaluated the efficacy of co-administering letrozole and clomiphene citrate (CC) in patients in POSEIDON group 4 with mild ovarian stimulation (OS). Although the difference was not statistically significant, the letrozole + CC group exhibited a higher LBR. However, this study faced a notable methodological challenge that may have affected the results: the utilization of two different pituitary suppression protocols, progestin-primed ovarian stimulation (PPOS) and GnRH antagonists. To elucidate the potential beneficial effect of letrozole on patients with expected POR, larger and higher-quality randomized controlled trials (RCTs) are warranted.

Previous studies have indicated that women in POSEIDON groups 3 and 4 typically experience lower LBR compared to normoresponders or those in POSEIDON group 1 and 2 [6]. Recommended strategies for achieving one euploid blastocyst in these expected POR patients include high-dose gonadotropin administration (300–450 IU/day), oocyte/embryo pooling, and duostim/luteal phase oocyte retrieval protocols [4,31]. However, it is crucial to acknowledge that these approaches may elevate treatment costs. The current study illustrates that letrozole administration can effectively reduce both the duration of stimulation and the total dose of gonadotropins required. This not only enhances the patient experience during a stressful treatment but also makes the treatment more economical. Özmen et al. [12] reported a reduction of approximately 6,000 US dollars in the total cost of achieving a clinical pregnancy with the use of letrozole. In addition, the latest meta-analysis demonstrated that letrozole administration resulted in significant reductions in both the total amount of gonadotropins consumed (by 506.72 IU; 95% CI, −803.27 to −210.16; *p* < 0.001) and the duration of follicle-stimulating hormone (FSH) stimulation (by 1.10 days; 95% CI, −1.54 to −0.66; *p* < 0.001) [23]. Our findings corroborate these results on dynamics of ovarian stimulation. Although it remains uncertain whether similar savings in treatment expenses can be realized presently, the utilization of letrozole still presents economic advantages to patients by diminishing the need for gonadotropins and shortening the duration of ovarian stimulation.

In this study, letrozole was administered for five days, and no significant difference was observed in the mean number of high-quality embryos obtained between the GnRH-ant and GnRH-LZ groups. However, Shapira et al. [32] reported that daily usage of 5 mg of letrozole from the first day of gonadotropin stimulation until the trigger day improved the quality of embryos in poor responders. It is worth noting that they did not use the POSEIDON classification system to define POR. The study analyzed only 24 women and did not report live birth rates. While they found an improvement in IVF outcomes with the extended use of letrozole, Fauda et al. [33] did not observe the same positive effect of letrozole in their evaluation of the extended use of letrozole with more patients. To the best of our knowledge, only two studies in the literature have evaluated the effect of letrozole use for more than five days. Based on the available data, it is not possible to draw definitive conclusions about the impact of extended letrozole use on ART outcomes. However, further research is needed to fully understand the efficacy of extended letrozole use, but future research in this area faces significant challenges due to safety concerns related to the teratogenic effects of letrozole. In an oral presentation by Biljan et al. [34] in 2005, letrozole use was associated with congenital cardiovascular anomalies and locomotor disabilities in offspring, but these findings were never published in a peer-reviewed journal as a research article. Subsequent extensive research has alleviated these concerns by demonstrating no such correlation [35]. However, neonatal outcomes after letrozole co-treatment are often inferred from studies focused on ovulation induction. It seems imperative that future studies evaluating the effects of extended letrozole use also include neonatal results alongside reproductive outcomes. This approach would greatly contribute to filling a notable gap in the scientific literature.

This study assessed the impact of letrozole on ART outcomes specifically in patients classified with expected POR according to the POSEIDON classification system. Patient inclusion criteria were based on antral follicle count and serum AMH levels, leading to a more uniform study population, although the retrospective study design has inherent limitations. Owing to the retrospective nature of the study, notable discrepancies emerged in the demographic characteristics and ovarian reserve markers among the included patients, thus constituting the primary limitation of the study. In addition, other limitations of the study were the absence of reporting cumulative live birth rates and neonatal outcomes. These parameters are crucial for providing comprehensive information to clinicians regarding the use of letrozole in ovarian stimulation. Future studies that incorporate these outcome measures will offer valuable insights into the efficacy and safety of letrozole administration in this context. Notably, the GnRH antagonist protocol was utilized alongside letrozole as the sole adjunctive medication, enabling a more precise evaluation of letrozole’s effect. In future studies, there is also a necessity for research focusing on the utilization of oral agents such as clomiphene citrate and letrozole independently in patients with poor ovarian response.

## 5. Conclusions

In conclusion, the administration of 5 mg/day of letrozole as an adjuvant medication for five days demonstrates notable reductions in the total amount of gonadotropin required, the duration of ovulation induction, and peak serum estradiol levels among patients with expected POR as per the POSEIDON classification system. However, this intervention did not yield significant effects on clinical pregnancy or live birth rates.

## Figures and Tables

**Table 1:** The comparison of demographic and cycle characteristics between groups.

	GnRH-ant Group (*n* = 281)	GnRH-LZ Group (*n* = 168)	*p*
Age (years)	34.9 ± 5.1	36.4 ± 4.9	0.002
Duration of infertility (years)	5.9 ± 5.1	7.5 ± 5.8	0.004
Number of previous IVF/ICSI treatments	1.1 ± 1.4	1.5 ± 1.8	0.028
Basal FSH (mIU/mL)	11.9 ± 7.5	13.7 ± 8.4	0.022
Basal estradiol (pg/mL)	57.6 ± 60.6	52.7 ± 46.5	0.338
AMH (ng/mL)	0.66 ± 0.5	0.56 ± 0.6	0.154
AFC, no.	3.9 ± 1.8	3.7 ± 1.8	0.006
Duration of ovarian stimulation (days)	11.2 ± 3.9	10.2 ± 3	0.005
Total dose of gonadotropins (IU)	3108.9 ± 1075.6	2661.3 ± 1284.5	<0.001
EMT on the day of trigger (mm)	10.4 ± 8.2	9.7 ± 1.8	0.247
Peak estradiol level (pg/mL)	913.4 ± 603.1	477.5 ± 487.9	<0.001
Number of follicles ≥ 17 mm on the day of trigger	2 ± 1.3	2.2 ± 1.5	0.199
Number of oocytes retrieved	2.7 ± 2	2.5 ± 2	0.399
Number of MII oocytes	2 ± 1.8	1.8 ± 1.4	0.198
Number of 2PN embryos	1.3 ± 1.2	1.3 ± 1.1	0.974
Number of high-quality embryos	0.5±0.8	0.4 ± 0.7	0.171
Number of transferred embryos	0.8 ± 0.7	1 ± 0.7	0.053
Fertilization rate, %	58.9	66.3	0.051
Implantation rate, %	13.3	8.3	0.066

The data are presented as the mean ± standard deviation. IVF, in vitro fertilization; ICSI, intracytoplasmic sperm injection; FSH, follicle-stimulating hormone; AMH, anti-Müllerian hormone; AFC, antral follicle count; EMT, endometrial thickness; MII, metaphase II; 2PN, 2 pronuclear. *p* < 0.05 was considered to indicate statistical significance. In neither group were there any patients diagnosed with ovarian hyperstimulation syndrome.

**Table 2:** The comparison of reproductive outcome characteristics between women in POSEIDON group 3 and group 4, with and without adjuvant letrozole administration.

	POSEIDON 3		*p*	POSEIDON 4		*p*
Outcome Parameter (Per Cycle)	GnRH-ant (*n* = 121)	GnRH-LZ (*n* = 56)		GnRH-ant (*n* == 160)	GnRH-LZ (*n* = 112)	
Clinical pregnancy rate	20 (16.4%)	8 (14.3%)	0.703	27 (16.9%)	11 (9.8%)	0.098
Miscarriage rate	6 (4.9%)	1 (1.8%)	0.313	10 (6.3%)	3 (2.7%)	0.174
Live birth rate	14 (11.6%)	7 (12.7%)	0.858	17 (10.6%)	8 (7.1%)	0.327

## Data Availability

Data available upon reasonable request from corresponding author due to privacy and ethical restrictions.
